# Dr. Reid, on the Warm Baths of Russia

**Published:** 1805-01-01

**Authors:** J. Reid

**Affiliations:** Southampton Row, Russell Square


### *To the Editors of the Medical and Phyfical Journal.*

GENTLEMEN,

THE subsequent question may appear to you sufficiently interesting and important to justify the insertion of some facts relating to it, in your highly respectable and widely extended publication.

How can it be explained that catarrh, phthisis, and rheumatism do not more prevail in Russia, as the inhabitants of this country are particularly exposed to great and abrupt changes of temperature? This is more particularly exemplified in their bagnios or warm-baths. These are either public public or private. In the public, persons are admitted by paying a certain stipend. The private, which are very numerous, are attached to the dwelling houses of opulent individuals. The public bagnios consist of a large room or rooms with elevated seats, the uppermost reaching nearly to the ceiling. These rooms are heated by means of an oven, having some large pebble-stones at the top over the fire; these become heated almost to a white-heat, then boiling water is thrown upon them; the room is soon filled with vapour heated in proportion to the quantity of water thrown upon the stones, and is seldom less than 140° of Fahrenheit's thermometer.

The common people are in the habit of going to these baths two days every week. These are called the public bathing days. Here you may see from two to three hundred persons, perfectly naked, without any regard to age or sex. After they have exposed themselves to the above heat from ten to thirty minutes or more, they come into an open court yard in the summer, pour cold water upon their heads and bodies, and in the winter plunge themselves into snow. On their return from the bath the whole surface of the body is of a very florid colour; which does not disappear either on the exposure to atmospherical air, the affusion of cold water, or any other means; but continues for some hours, diminishing in a slow and gradual manner.*

The air of Russia is generally dry; the changes are very sudden, and the extremes great. Their winters are much colder than ours in England. It is not uncommon for the thermometer of Fahrenheit to be from 40° to 50° below the freezing point during a considerable period; at the same time that their summers are much hotter than ours.

A much respected and esteemed friend, Mr. Taunton, of Pater-noster Row, has communicated to me the results of his own personal experience with regard to the baths in Russia.

"June 28, 1803, three A. M. I went into the bath with my clothes on, heated to 45° of Reaumur's scale, equal to 133 of Fahrenheit, pulse 56; when I had been in twenty-three minutes it increased to 150, and afterwards became so quick as not to be numbered; profuse perspiration from every part of the body; respiration not much altered; a continued stream of water ran from the mouth and lungs. On

* See Fordyce's Dissertation on Fever, p. 168—9.

On going into the air a slight sickness came on, and continued for some hours with a little difficulty of breathing. Great debility remained during the day, with a sensation of cold. Did not sleep at night, though apparently overpowered by it in the afternoon; the perspiration appeared to cease instantly on exposure to the air, (which was cool for the season) but a general softness came on the skin after walking about half a mile.

"June 30, 1803, six P. M. I went into the bath (first taking off my clothes) heated to 55° of Reaumur, equal to 156 of Fahrenheit; pulse 60; rose in ten minutes to 152, and in five minutes more became so quick as not be numbered. A violent smarting sensation was produced on the skin; respiration not much affected, but rather quicker than natural; dressed and walked into the air immediately; perspiration appeared checked, but took no cold, and did not get any sleep during the night."

I hope to receive from some ingenious correspondent an explanation with regard to the question suggested in this communication. If I should be disappointed, I shall v[illegible]nture to send you in a little time my own conjectures, in relation to this curious and important subject.

I am, &c.

*Southampton Row, Russell Square,* J. REID.

